# Profile of patients referred to the active rehabilitation unit in the health area of Soria (Spain)

**DOI:** 10.1192/j.eurpsy.2022.1945

**Published:** 2022-09-01

**Authors:** R. Martínez Gallardo, N. Palomar Ciria, B. Valiente Peña, N. Hernandez Vicente

**Affiliations:** 1 Complejo Hospitalario de Soris, Psiquiatra, Soria, Spain; 2 Complejo Hospitalario, Psiquiatría, Soria, Spain

## Abstract

**Introduction:**

Realizamos un estudio prospectivo en 15 pacientes, diagnosticados de psicosis, según el DSM V, y que son usuarios de una Unidad de Rehabilitación activa, estos pacientes utilizan las modalidades de hospitalización residencial o el programa de hospitalización parcial.

**Objectives:**

El objetivo principal fue establecer el perfil del paciente (retrato de robot), que utiliza este tipo de dispositivo.

**Methods:**

Se lleva a cabo un protocolo de evaluación que incluye la entrevista de evaluación psicopatológica (DSMV) y las escalas: impulsividad (Barrat), agresividad (Burke), calidad de vida (Woqol bref ), actitud ante la medicación (DAI), Avd ( RAI), riesgo de suicidio (Plutchick), riesgo de caídas (Downton), funcionalidad (EEAG) y hábitos de consumo (DAU).

**Results:**

Con todos estos elementos y teniendo en cuenta la edad y sexo de los pacientes, intentamos establecer el tipo de perfil de paciente del área de Rehabilitación activa de nuestro servicio, luego luego de mediciones periódicas de todas estas variables estableceremos la influencia de Terapia de rehabilitación en la mejora o empeoramiento de nuestros pacientes.

**Conclusions:**

CONCLUSIONES El perfil de tipo del paciente incluido en una Unidad de Rehabilitación Activa está compuesto por un varón de 42 años, con consumo esporádico de toxinas, con rasgos de personalidad donde predomina la agresividad, con un perfil bajo de efectos adversos y con conciencia parcial de la enfermedad. y mala adherencia a la medicación Cabe señalar que sus niveles en la escala de calidad de vida son altos, incluso comparables o en ocasiones superiores a los de la población general según la escala Woqol bref.

**Disclosure:**

No significant relationships.

